# Optimal vehicle size and driving condition for extended-range electric vehicles in China: A life cycle perspective

**DOI:** 10.1371/journal.pone.0241967

**Published:** 2020-11-20

**Authors:** Yongtao Liu, Jie Qiao, Haibo Xu, Jiahui Liu, Yisong Chen

**Affiliations:** School of Automobile, Chang’an University, Xi’an, China; Nanyang Technological University, SINGAPORE

## Abstract

Many researchers use life cycle assessment methodology to investigate the energy and environmental impacts of energy-saving and new energy vehicles. However, in the context of China, the life cycle energy-saving and emission-reduction effects of extended-range electric vehicles (EREVs), and the optimal applicable vehicle size and driving conditions for EREVs have been rarely studied. In this study, based on the life cycle assessment theory, the resource consumption, energy exhaustion, and environmental impact of EREVs were comprehensively analyzed. In addition, a differential evaluation model of ecological benefits was established for comparing EREVs with other vehicles with different power sources. Finally, scenario analysis was performed in terms of different vehicle sizes and driving conditions. The results have shown that EREV has great advantages in reducing mineral resource consumption and fossil energy consumption. The consumption of mineral resources of EREV is 14.68% lower than that of HEV, and the consumption of fossil energy is 34.72% lower than that of ICEV. In terms of environmental impact, EREV lies in the middle position. The scenario analysis has revealed that, for EREV in China, the optimal vehicle size is the passenger car and the optimal driving condition is the suburban condition. This work helps to understand the environmental performance of EREVs in China and may provide a decision-making reference for the government.

## 1. Introduction

Energy-saving and new energy vehicles are recognized as one of the most important development trends for the future automobile, featuring reducing energy consumption and emissions. Many researchers use life cycle assessment methodology to investigate the energy and environmental impacts of energy-saving and new energy vehicles. As a vehicle model that transitions from a fuel-powered vehicle to a pure electric vehicle, the extended-range electric vehicle introduces a range extender so that the engine does not directly participate in power transmission, but supplements the power battery with electric energy. With this working principle, the engine can always be maintained within the best fuel efficiency area, and the system efficiency can be effectively improved, thus better energy saving and emission reduction effects can be achieved. However, the specific energy-saving and emission-reduction effect of the extended-range electric vehicle in the full life cycle, the optimal applicable vehicle type and operating conditions for the extended-range electric vehicle, as well as the advantages and disadvantages of the extended-range electric vehicle compared with other power source models need to be thoroughly studied.

### 1.1 Literature review

Many scholars have studied the energy consumption and environmental characteristics of different types of new energy vehicles, including battery electric vehicles (BEVs) [1, 2], hybrid electric vehicles (HEVs) [3, 4], plug-in hybrid electric vehicles (PHEVs) [5], fuel cell vehicles (FCVs) [6, 7], natural gas vehicles (NGV) [8, 9], extended-range electric vehicles (EREVs) [10]. Particularly, Bohnes et al. explored relative environmental benefits of EREVs and FCVs over internal combustion engine vehicles (ICEVs) and electric vehicles [11]. Karabasoglu et al. studied the impact of driving conditions on the economic and environmental benefits of electric vehicles and found that in New York’s driving cycle, hybrid and extended-range plug-in hybrid can reduce the life-cycle emissions by 60% and costs by 20% compared to the conventional vehicles [12].

Moreover, some scholars conducted comparative studies on life cycle energy consumption and emissions of vehicles with different power sources [13–16]. For example, Shi et al. evaluated the energy saving and emission reduction potential of BEVs by comparing traditional gasoline and battery electric vehicles [17]. Hooftman et al. studied the environmental impacts of vehicles with three different power systems, i.e., PHEV, BEV with a battery capacity of 40 kWh, and long-range electric vehicles with 90 kWh [18]. Zhao et al. analyzed the greenhouse effect of different dynamic types of electric trucks in the whole life cycle [19].

In addition, the environmental impacts of the vehicle at different life cycle stages were investigated. Qiao et al. found that the production stage of electric vehicles emits 14.6–14.7 tons of CO_2_, which was 59%-60% higher than ICEVs [20]. Kawamoto et al. demonstrated that the battery production stage generates a large amount of CO_2_. During the assembly stage, BEVs generate larger amount of CO_2_ than ICEVs. But in the regions where renewable energy is widely used for power generation, as vehicle mileage increases, BEVs have less CO_2_ emissions than ICEVs [21]. Bickert et al. found that although BEVs have larger amount of CO_2_ emissions than ICEVs in the production stage, the CO_2_ emissions from BEVs can be reduced by travelling 2500–5500 km/day during the operation stage [22].

Also, some studies have considered the effects of actual driving conditions, mileage, and vehicle size on life cycle energy consumption and emissions. Yuan et al. found that, BEVs have a great energy-saving advantage in the driving cycle of low speed and frequent parking, whereas when driving at high speeds, BEVs tend to have higher energy consumption [23]. Karabasoglu et al. found that in the urban driving cycle in New York City, hybrid and extended-range plug-in hybrid vehicles can reduce life cycle emissions by 60% and costs (CVs) by 20% compared to conventional vehicles [24]. Correa et al. compared the energy and environmental performance of vehicles and demonstrated that HEVs have the best effect in the short/medium period. But in the long run, BEVs are suitable for shorter mileage, while fuel cell buses are suitable for longer mileage [25]. In terms of vehicle size, Yang et al. found that plug-in electric trucks have better performance in cost and greenhouse gas emissions than light/medium diesel trucks and replaceable battery electric trucks [26], while Faria et al. conducted environmental and economic life cycle assessments of conventional and electric vehicle technologies, testing the compact and mini vehicles in practical scenarios [27].

However, in the context of China, the life cycle energy-saving and emission-reduction effects of EREVs, the optimal applicable vehicle size and driving conditions for EREVs, as well as the advantages and drawbacks of EREVs compared with other types of powertrain are rarely studied.

### 1.2 Contribution of this work

To bridge this research gap, this work focuses on Chinese context and explores the life cycle energy and environmental effects of EREVs, the optimal applicable vehicle size and driving conditions for EREVs, as well as the pros and cons of EREVs compared with other types of powertrain. First of all, this paper uses the full life cycle method (including the vehicle cycle and the fuel cycle) to quantitatively analyze the effects of energy saving and emission reduction of EREVs. Then, EREVs are compared with other five types of energy-saving and new energy vehicles with the same vehicle size. In addition, a comparative analysis of three different vehicle sizes of EREVs is performed. Moreover, six different types of vehicles in different driving conditions are analyzed.

This work might help to understand the environmental performance of EREVs in China and provide a basis for ecological design, green manufacturing, and full life cycle management for EREVs. Also, this work may provide a theoretical support and decision-making reference for the government or relevant functional departments responsible for formulating relevant policies or regulations for energy-saving and new energy vehicles.

## 2. Methods

### 2.1 Research object and system boundary

In this study, an ordinary sedan EREV, featuring mature technology, large market retention, and extensive utilization area, is used as the research object to ensure the typical representative. Table 1 shows the basic performance parameters of the research object.

**Table 1:** Basic performance parameters of EREV.

Main parameters	Whole vehicle mass (kg)	Engine capacity (L)	Maximum engine power (kw)	Total motor power (kw)	Battery type	Battery capacity (kWh)
Parameter value	1700	1.4	63	111	Lithium battery	16

^1^Note: The parameter values are obtained from official data.

**Fig 1:**
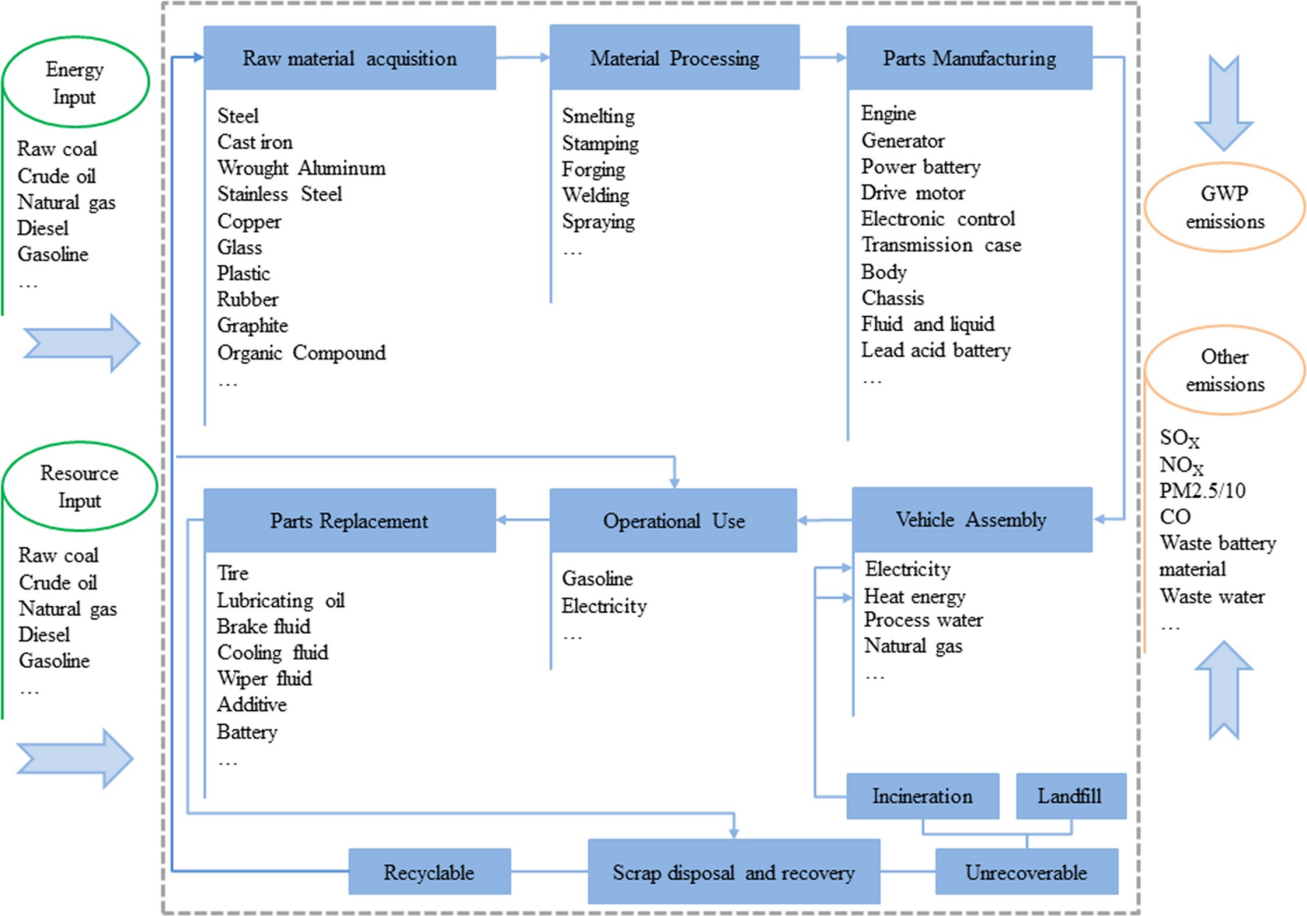
System boundary for EREV.

Fig 1 shows the system boundary diagram for the full life cycle evaluation of EREVs. The life cycle is divided into four stages: raw material acquisition stage, manufacturing and assembly stage, operation stage, and disposal and recycling stage. Three types of non-renewable energy are mainly considered, i.e., raw coal, crude oil, and natural gas. The following environmental emission pollutants are mainly considered, such as CO_2_, CO, NO_X_, SO_X_, CH_4_, PM2.5, PM10, and NMVOC. According to the characteristics of EREVs, EREV is divided into four parts including the main body of the car, the engine, the lithium-ion battery, and the fluid. The main body of the car can be further divided into six parts, i.e., body, main speed reducer, chassis, motor, generator and electric control devices. Some materials or parts with small mass or little influence on the evaluation results are replaced or omitted.

### 2.2 Functional units and data sources

The functional unit refers to the quantified product function or performance characteristics [28]. At present, there is a general consensus on the life cycle mileage of conventional vehicles, which is around 15,000–300,000 km [29]. However, the life cycle mileage of energy-saving and new energy vehicles is affected by battery life, stack life, the fact that the market input period has not reached the end of the life cycle, etc., resulting in a large difference in the data. From the official data, the warranty of EREV covers three years or 100,000 kilometers. According to the literature [30, 31] and the actual market usage, a driving distance of 150,000 km of EREV travelling under a comprehensive operating condition in China was considered as the functional unit.

In this paper, the actual data (data of vehicle parts list) at each stage of the EREV are partly from the references in China and other countries [32–36], and partly from the enterprise research. The basic data mainly refer to the basic energy consumption of metal materials and automotive energy, the basic data of gas emissions as well as toxic and hazardous substances of automotive energy, the basic data of alloy materials and non-metallic materials, etc. The basic data stem from CLCD database, European Ecoinvent database, GaBi7 software database, etc.

### 2.3 Life cycle assessment model of EREV

The vehicle material mass of EREV is determined by the matrix *M* = [*m*_*i*_]_1×*k*_, in which m_*i*_ is the mass of the ith raw material in the vehicle. The utilization matrix in the component processing process is *α* and the conversion rate matrix in the material preparation process is *β*, then the EREV life cycle material consumption is given by Eq (1).

$$M_{f}=M•\alpha^{-1}•\beta^{-1}$$ (1)

The EREV life cycle energy consumption *E* is calculated by determining the energy consumption at each stage.

$$E=E_{acq}+E_{man}+E_{use}+E_{rec}$$ (2)

where *E*_*acp*_ = [*e*_m*ij*_]_×*b*_, e_*mij*_ is the jth energy consumption by the ith raw material. *E*_man_ = [*e*_*pij*_]_×*b*_, e_*pij*_ is the jth energy consumption by the processing and manufacturing of the ith component *E*_use_ = *F*•*E*_*F*_. *E*_use_ = *F*•*E*_*F*_, *F* is the fuel consumption matrix, *E*_*F*_ is the matrix of various energy consumptions to produce the unit fuel *E*_*rec*_ = *M*_m_•*E*_*neg*_−*M*_m_•*E*_pos_. *E*_*rec*_ = *M*_m_•*E*_*neg*_−*M*_m_•*E*_pos_, *M*_*m*_ is the material matrix at the disposal stage, *E*_*neg*_ is the energy consumption matrix per unit mass of recycled materials, *E*_pos_ is the recovered energy per unit mass of recycled materials.

The full life cycle emissions of EREV are determined by the emissions at various stages.

$$P=P_{acq}+P_{man}+P_{use}+P_{rec}$$ (3)

where *P*_acq_ = *M*•[p_*manij*_]_*k*×*u*_, p_*acqij*_ is the emission intensity of jth pollutant to obtain the ith material. *P*_*man*_ = [*m*_*comi*_]_1×*p*_•[*p*_*mainij*_]_*p*×*u*_, m_*comi*_ is the mass of the ith component of the vehicle, *p*_*mainij*_ is the emission intensity of the jth pollutant during the manufacturing process of the ith component. *P*_*use*_ = *F*•*P*_*F*_, *P*_*F*_ is the pollutant emission matrix per unit of consumed fuel. *P*_*rec*_ = *M*_*m*_•*P*_*neg*_−*M*_*m*_•*P*_*pos*_, *P*_neg_ is the pollutant emission matrix for recycling unit mass materials, *P*_*pos*_ is the pollutant reduction matrix for recycling unit mass materials.

### 2.4 Differential evaluation model of ecological benefits of vehicles with different power sources

The differential evaluation model of the ecological benefits of vehicles with different power sources is designed, in order to calculate the differences in mineral resource depletion, fossil energy depletion, and environmental impact between vehicle A with the power source of PP_Ⅰ_ and vehicle B with the power source of PP_Ⅱ_ (The size of vehicle A and vehicle B remain the same).

#### 2.4.1 Resource depletion differential evaluation model

1) Differential model of material consumption

(1) Differential matrix of vehicle mass

The number of components, material types, and masses of vehicle A and vehicle B are determined respectively, and the initial vehicle mass matrices of vehicle A and vehicle B, i.e., $M_{IA}={\left(m_{aij}\right)}_{p_{1}\times q_{1}}$ and $M_{IB}={\left(m_{bkr}\right)}_{p_{2}\times q_{2}}$, are constructed, respectively. In the matrices, *p*_1_ and *p*_2_ represent the number of components in vehicle A and vehicle B, respectively; *q*_1_ and *q*_2_ represent the number of material types contained in vehicle A and vehicle B, respectively; and *m*_*a*_(*m*_*b*_) indicates the mass of the j(r) type of material contained in the j(k) component in the vehicle A (B).

During the life cycle of the vehicle, some parts need to be replaced (such as tires, etc.), thus the part replacement matrices for vehicle A and vehicle B are constructed, i.e., $F_{A}=diag\left\{fa_{11},fa_{22},\cdots ,fa_{p_{1}p_{1}}\right\}$, $F_{B}=diag\left\{fb_{11},fb_{22},\cdots ,fb_{q_{2}q_{2}}\right\}$, where *fa*_*ii*_ and *fb*_*kk*_ represent the replacement times of the ith type of parts in vehicle A and the kth type of parts in vehicle B during the life cycle, respectively. Therefore, considering the replacement of parts, the life cycle total mass matrices of vehicle A and vehicle B are *M*_*TA*_ = (*E*+*F*_*A*_)•*M*_*IA*_ and *M*_*TB*_ = (*E*+*F*_*B*_)•*M*_*IB*_, respectively, where *E* is the unit diagonal matrix.

Vehicle A and vehicle B have different power sources, and the number of parts and material types are not exactly same. Assume that the total number of part types in vehicle A and vehicle B is *e*, and the total number of material types in vehicle A and vehicle B is *h*, then the mass differential matrix *M*_*TD*_ of vehicle A and vehicle B *M*_*TD*_ is as follows,

$$M_{TD}=\left[\begin{array}{ccccc} m_{td11} & m_{td12} & \cdots & \cdots & m_{td1h}\\ \vdots & \vdots & & & \vdots \\ m_{tds1} & m_{tds2} & \cdots & \cdots & \vdots \\ \vdots & \vdots & & & \vdots \\ m_{tdr1} & m_{tdr2} & \cdots & \cdots & \vdots \\ \vdots & \vdots & & & \vdots \\ m_{tde1} & m_{tde2} & \cdots & \cdots & m_{tdeh} \end{array}\right]$$ (4)

where *s* is the number of the part types unique for vehicle A (not contained in vehicle B), *e*−*r* is the number of the part types unique for vehicle B (not contained in vehicle A), *r*−*s* is the number of common parts in vehicle A and vehicle B, *m*_*tdij*_ indicates additional mass of the jth material consumed by vehicle A than vehicle B when preparing the ith part.

(2) Differential matrix of mineral resource consumption

The preparation of the materials for the parts can be divided into two main processes. Firstly, the raw materials (ore, crude oil, raw coal, etc.) are used to produce the basic materials such as iron, aluminum, plastics, and rubber through smelting and chemical reaction. Secondly, the basic materials are processed into the vehicle materials through turning, milling, planning, cutting, etc.

The mass difference matrix of vehicle A and vehicle B is converted into the mass difference matrix for the hth material of vehicle A and vehicle B, $M_{TD}^{Ⅲ}={\left(\sum_{i=1}^{e}m_{tdij}^{Ⅲ}\right)}_{1\times h}={\left(m_{tdj}^{Ⅲ}\right)}_{1\times h}$. $m_{tdj}^{Ⅲ}$ is obtained by summing the jth column of *M*_*TD*_, which represents the mass difference of the jth material consumed in the production of parts.

According to the analysis results of the list, the utilization matrix of the basic materials in the part processing process is *η* = *diag*{*η*_11_,*η*_22_,⋯,*η*_*jj*_,⋯,*η*_*hh*_}, and the utilization matrix of the raw materials during the material production process is *φ* = (*φ*_*ij*_)_*z*×*h*_. When the basic materials are processed into automotive materials, there is no change in the material composition, thus *η* is a diagonal matrix. *η*_*ii*_ indicates the utilization rate of the jth type of materials during the processing. During the production process of the basic materials from the raw materials, the types of materials have changed. *z* is the type of raw materials and *φ*_*ij*_ is the utilization rate of the ith type of raw materials in the production process of the jth type of basic materials.

The differential matrix of the basic material consumption mass of vehicle A and vehicle B is as follows,

$$M_{TD}^{Ⅱ}=M_{TD}^{Ⅲ}\cdot \eta^{-1}$$ (5)

The differential matrix of the mineral resources (raw materials) consumption mass of vehicle A and vehicle B is as follows,

$$M_{TD}^{Ⅰ}=M_{TD}^{Ⅲ}•\eta^{-1}•\varphi^{-1}$$ (6)

2) Differential model of energy consumption

(1) Raw material acquisition stage

The energy intensity matrix for energy production is constructed to be *E*_*O*_ = (*e*_*oij*_)_*a*×*b*_, where *e*_*oij*_ represents the consumption of the jth type of primary energy (raw coal, crude oil, natural gas, etc.) to produce per unit of the ith type of secondary energy (electricity, heat, etc.), *a* represents the number of secondary energy types, *b* represents the number of primary energy types.

The energy consumption intensity matrix is $E^{Ⅱ}={\left(e_{ij}^{Ⅱ}\right)}_{h\times a}$ at the basic material production stage and $E^{Ⅲ}={\left(e_{ij}^{Ⅲ}\right)}_{h\times a}$ at the vehicle material production stage, respectively. $e_{ij}^{Ⅱ}$ represents the consumption of the jth type of second secondary energy to produce per unit mass of the ith type of basic material, and $e_{ij}^{Ⅲ}$ represents the consumption of the jth type of second secondary energy to produce per unit mass of the ith type of vehicle material.

Therefore, the energy consumption differential matrix of vehicle A and vehicle B at the raw material acquisition stage is as follows,

$$E_{acq-D}=M_{TD}^{Ⅱ}•E^{Ⅱ}•E_{O}+M_{TD}^{Ⅲ}•E^{Ⅲ}•E_{O}$$ (7)

(2) Manufacturing and assembly stage

The energy consumption matrix for the manufacturing of automotive parts from the vehicle materials is *E*_*man*_ = (*e*_*manij*_)_*p*×*a*_ and the energy consumption matrix during the assembly process of the parts is *E*_*ass*_ = (*e*_*assij*_)_*p*×*a*_. *e*_*manij*_ is the consumption of the jth type of secondary energy to manufacture the ith type of auto parts, and *e*_*assij*_ is the consumption of the jth type secondary energy to assemble the ith type of auto parts, in which the values of parameter *p* are *p*_1_ and *p*_2_ for vehicle A and vehicle B, respectively.

Therefore, the energy consumption differential matrix between vehicle A and vehicle B during the manufacturing and assembly stage *E*_*maa*−*D*_ is shown in Eq (8).

$$\begin{array}{l} E_{maa-D}=\left[{\left(e_{manij}^{A}\right)}_{p_{1}\times a}+{\left(e_{assij}^{A}\right)}_{p_{1}\times a}-{\left(e_{manij}^{B}\right)}_{p_{2}\times a}-{\left(e_{assij}^{B}\right)}_{p_{2}\times a}\right]•E_{O}\\ \;=\left(E_{man}^{A}+E_{ass}^{A}\right)•E_{O}-\left(E_{man}^{B}+E_{ass}^{B}\right)•E_{O} \end{array}$$ (8)

(3) Operation stage

In order to compare the energy consumption and emissions of vehicle A and vehicle B throughout the life cycle, it can be assumed that the total mileages of vehicle A and vehicle B are both L, and the total fuel consumption per unit mileage (gasoline, diesel, hydrogen, electricity, etc.) is *Q* = *Q*_*gas*_+*Q*_*die*_+*Q*_*hyd*_+*Q*_*ele*_. *Q*_*gas*_, *Q*_*die*_, *Q*_*hyd*_, and *Q*_*ele*_, representing the amount of gasoline, diesel, hydrogen, and electricity consumed by the vehicle per unit mileage, respectively. The amounts and types of fuel consumed by vehicles with different power sources are different.

The energy consumption matrices for the energy consumption of gasoline, diesel, hydrogen, and electricity are *E*_*gas*_ = (*e*_*gasj*_)_1×*a*_, *E*_*die*_ = (*e*_*diej*_)_1×*a*_, *E*_*hyd*_ = (*e*_*hydj*_)_1×*a*_, and *E*_*ele*_ = (*e*_*elej*_)_1×*a*_, respectively, in which *e*_*gasj*_, *e*_*diej*_, *e*_*hydj*_, and *e*_*elej*_ represent the consumption of the ith type of primary energy to produce per unit of gasoline, diesel, hydrogen, and electricity.

Therefore, the energy consumption differential matrix *E*_*use−D*_ between vehicle A and vehicle B at operation stage *E*_*use−D*_ is as follows,

$$E_{use-D}=\left[\left(Q_{gas}^{A}-Q_{gas}^{B}\right)E_{gas}+\left(Q_{die}^{A}-Q_{die}^{B}\right)E_{die}+\left(Q_{hyd}^{A}-Q_{hyd}^{B}\right)E_{hyd}+\left(Q_{ele}^{A}-Q_{ele}^{B}\right)E_{ele}\right]$$ (9)

(4) Disposal and recycling stage

There is a positive energy consumption (the energy generated by vehicle disassembly, crushing, cleaning, remanufacturing, etc.) and negative energy consumption (the recycled energy generated by reusable, reusable, remanufactured parts and materials, etc.). The positive energy consumption matrix and negative energy consumption matrix in the vehicle recycling process of vehicle A and vehicle B are constructed as follows: $E_{rec}^{A+}={\left(e_{recij}^{A+}\right)}_{p_{1}\times a}$, $E_{rec}^{A-}={\left(e_{recij}^{A-}\right)}_{p_{1}\times a}$, $E_{rec}^{B+}={\left(e_{recij}^{B+}\right)}_{p_{2}\times a}$, and $E_{rec}^{B-}={\left(e_{recij}^{B-}\right)}_{p_{2}\times a}$, respectively. $e_{recij}^{A+}$ and $e_{recij}^{B+}$ represent the consumption of the jth type of secondary energy to dismantle and recycle the ith type of part, respectively. $e_{recij}^{A-}$ and $e_{recij}^{B-}$ represent the indirect recovery of the jth type of secondary energy by recycling the ith type of part. The energy consumption differential matrix *E*_*rec−D*_ of vehicle A and vehicle B at the disposal and recycling stage *E*_*rec−D*_ can be expressed as shown in Eq (10).

$$E_{rec-D}=\left[\left(E_{rec}^{A+}-E_{rec}^{A-}\right)-\left(E_{rec}^{B+}-E_{rec}^{A-}\right)\right]•E_{O}$$ (10)

#### 2.4.2 Differential evaluation model of environmental impact

During the raw material acquisition stage, manufacturing and assembly stage, operation stage, and disposal and recycling stage of the vehicle, the energy consumption process such as combustion, welding, and smelting can generate emissions (solids, liquids, gases), which are called direct emissions. However, there are also emissions in the process of primary energy acquisition, which is called indirect emissions. Therefore, the pollutant emission intensity matrix at the energy acquisition stage is firstly constructed as $P_{O}^{E}={\left(p_{Oij}^{E}\right)}_{b\times u}$, where $p_{Oij}^{E}$ represents the emission intensity of the jth type of pollutant in the process of acquiring the ith type of primary energy, *b* is the number of primary energy types, and *u* is the total number of emission types in the vehicle life cycle. When the number of emission types is smaller than *u* at certain stage, the rest terms of the emission intensity matrix are filled with zeros.

*1) Raw material acquisition stage*. The acquisition emission intensity matrix of per unit mass material at the raw material acquisition stage is constructed as *P*_*acq*_ = (*p*_*acqij*_)_*h*×*u*_, where *p*_*acqij*_ represents the emission intensity of the jth type of pollutant in the process of acquiring per unit mass of the ith material. Therefore, at the raw material acquisition stage, the environmental impact differential matrix *P*_*acq−D*_ of vehicle A and vehicle B *P*_*acq−D*_ is given by Eq (11).

$$P_{acq-D}=M_{TD}•P_{acq}+E_{acq-D}•P_{O}^{E}$$ (11)

*2) Manufacturing and assembly stage*. The emission intensity matrix during the processing and manufacturing of automotive materials into parts is constructed as *P*_*man*_ = (*p*_*manij*_)_*p*×*u*_, and the emission intensity matrix during the assembly process of parts is constructed as *P*_*ass*_ = (*p*_*assij*_)_*p*×*u*_. *p*_*manij*_ is the emission intensity of the jth pollutant during the processing and manufacturing of the ith part, and *p*_*assij*_ is the emission intensity of the jth pollutant during the assembly process of the ith part. The value of *p* is set to *p*_1_ and *p*_2_ for vehicle A and vehicle B, respectively. Therefore, the environmental impact differential matrix of vehicle A and vehicle B at the manufacturing and assembly stage *P*_*maa−D*_ can be expressed as shown in Eq (12).

$$\begin{array}{l} P_{maa-D}=\left[{\left(p_{manij}^{A}\right)}_{p_{1}\times a}+{\left(p_{assij}^{A}\right)}_{p_{1}\times a}-{\left(p_{manij}^{B}\right)}_{p_{2}\times a}-{\left(p_{assij}^{B}\right)}_{p_{2}\times a}\right]+E_{maa-D}•P_{O}^{E}\\ \;=\left(P_{man}^{A}+P_{ass}^{A}\right)-\left(P_{man}^{B}+P_{ass}^{B}\right)+E_{maa-D}•P_{O}^{E} \end{array}$$ (12)

*3) Operation stage*. The emission intensity matrices for the production of gasoline, diesel, hydrogen, and electricity are constructed as *P*_*gas*_ = (*p*_*gasj*_)_1×*u*_, *P*_*die*_ = (*p*_*diej*_)_1×*u*_, *P*_*hyd*_ = (*p*_*hydj*_)_1×*u*_, and *P*_*ele*_ = (*p*_*elej*_)_1×*u*_, respectively. *p*_*gasj*_、*p*_*diej*_, *p*_*hydj*_, and *p*_*elej*_ represent the emission intensities of the jth type of pollutants consuming per unit of gasoline, diesel, hydrogen, and electricity, respectively. Therefore, the environmental impact differential matrix of vehicle A and vehicle B at the operation stage *P*_*use−D*_ can be given by Eq (13).

$$P_{use-D}=\left[\left(Q_{gas}^{A}-Q_{gas}^{B}\right)P_{gas}+\left(Q_{die}^{A}-Q_{die}^{B}\right)P_{die}+\left(Q_{hyd}^{A}-Q_{hyd}^{B}\right)P_{hyd}+\left(Q_{ele}^{A}-Q_{ele}^{B}\right)P_{ele}\right]L+E_{use-D}•P_{O}^{E}$$ (13)

*4) Disposal and recycling stage*. The differential matrix of the emission intensity at the disposal and recycling stage *P*_*rec−D*_ is calculated from the energy consumption in that process.

$$P_{rec-D}=\left[\left(E_{rec}^{A+}-E_{rec}^{A-}\right)-\left(E_{rec}^{B+}-E_{rec}^{A-}\right)\right]•E_{O}•P_{O}^{E}=E_{rec-D}•P_{O}^{E}$$ (14)

## 3. Results and discussion

### 3.1 Life cycle impact assessment of EREV

Currently, the life cycle impact assessment methods mainly include CML96, CML2001, Ecoindicator95, Ecoindicator99, Edip97, Edip2003, Traci, and Epfl2002+. Among them, the CML method developed by the CML laboratory of Leiden University in the Netherlands has been widely used. It divides environmental impact into 11 categories, i.e., mineral resource depletion potential (ADP element), fossil energy depletion potential (ADP fossil), global warming potential (GWP), acidification potential (AP), water eutrophication potential (EP), photochemical smog potential (POCP), ozone depletion potential (ODP), and human health damage potential (HTP). Based on the characteristics of China's local energy and environment and the generality of the evaluation method, we chose CML2001 as the evaluation method and utilized the first 7 types of impact indicators for the study. The GaBi software system platform, developed by the University of Stuttgart in Germany, has maturely implemented CML2001 impact evaluation method. Therefore, the GaBit software was used in this study to establish the evaluation model.

The life cycle assessment method is mainly to quantify, analyze and evaluate the mineral resources, fossil energy, and environmental emissions generated during the entire life cycle of the target object from raw material acquisition to scrap recycling. Many calculations involved in the life cycle assessment of new energy vehicles in this paper are all completed through GaBi software. The basic elements include target balance, plan, process, flow, project, quality indicator, weight and global parameters.

Parameters are mainly divided into original parameters and process parameters, which are the basis for advanced analysis (sensitivity analysis, Monte Carlo analysis, parameter change, and scenario analysis) in the software.

When software works, the research objectives and scope should be determined in the first step. Then, the inventory analysis is done to analyze a product system or activity in its entire life cycle. Data-based objective quantification of material consumption, energy consumption and environmental emissions at different stages is the basis for life cycle impact assessment. The list of research objects is input into GaBi for modeling to carry out the life cycle impact assessment, and the result can be got through the step of classification, feature words, normalization, quantification and other stages.

The specific calculation results are shown in Section 3.1.1.

#### 3.1.1 Analysis of energy consumption and emission results

**Fig 2:**
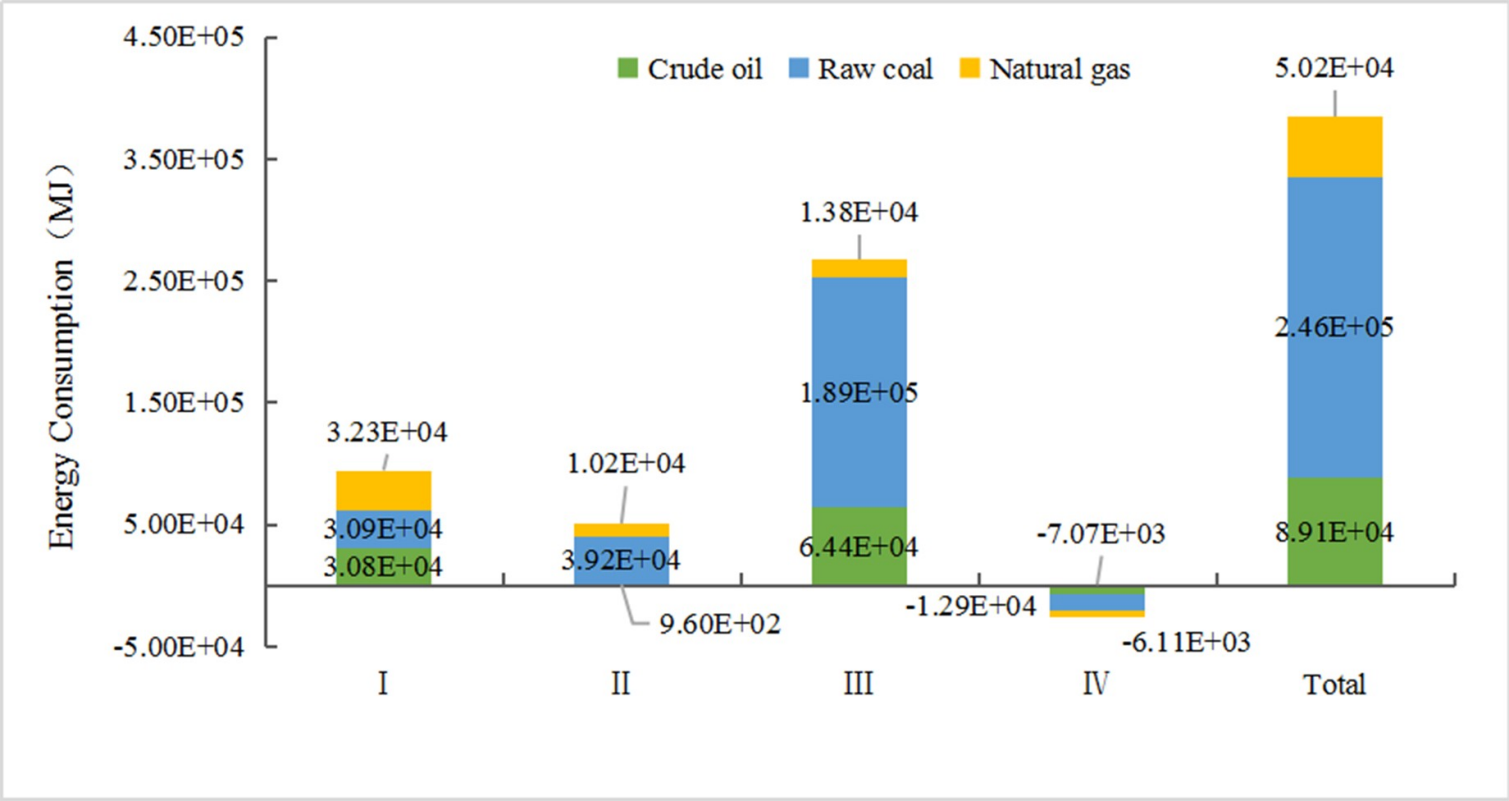
Energy consumption at each stage of EREV life cycle. (Note: I—Raw material acquisition stage, II—Manufacturing and assembly stage, III—Operation and use stage, IV—Scrap recovery stage).

In terms of energy consumption, this study mainly considers the consumption of non-renewable energy (raw coal, crude oil, natural gas) at various stages of the life cycle, but does not consider the depletion of renewable resources. In terms of emissions, this study mainly considers the major emissions with high environmental impacts. Fig 2 shows the calculation results of the energy consumption of the full life cycle of an EREV. During the life cycle, raw coal and crude oil are mostly consumed, of which the raw coal consumption is the largest, accounting for 63.85% of the total energy consumption. The main reason for the large consumption of coal is that the smelting process of mineral resources requires the combustion of a large amount of raw coal to generate heat, and at the same time, a large amount of electricity is consumed during the entire life cycle. In China, thermal power generation accounts for 70.39% of the current energy structure [37], and raw coal is the main raw material used for electricity generation. The consumption of crude oil mainly occurs at the raw material acquisition stage and the vehicle operation stage. In the raw material acquisition stage, crude oil is mainly used to provide power for mining and transportation of various mineral machinery and vehicles. At the same time, crude oil is also used as a raw material for the production of plastics and other chemical products. Crude oil is the mostly consumed at the operation stage. The EREV uses the gasoline as fuel, which is obtained through the processing of crude oil. Therefore, crude oil is mainly consumed in the form of a power source at the operation stage.

**Fig 3:**
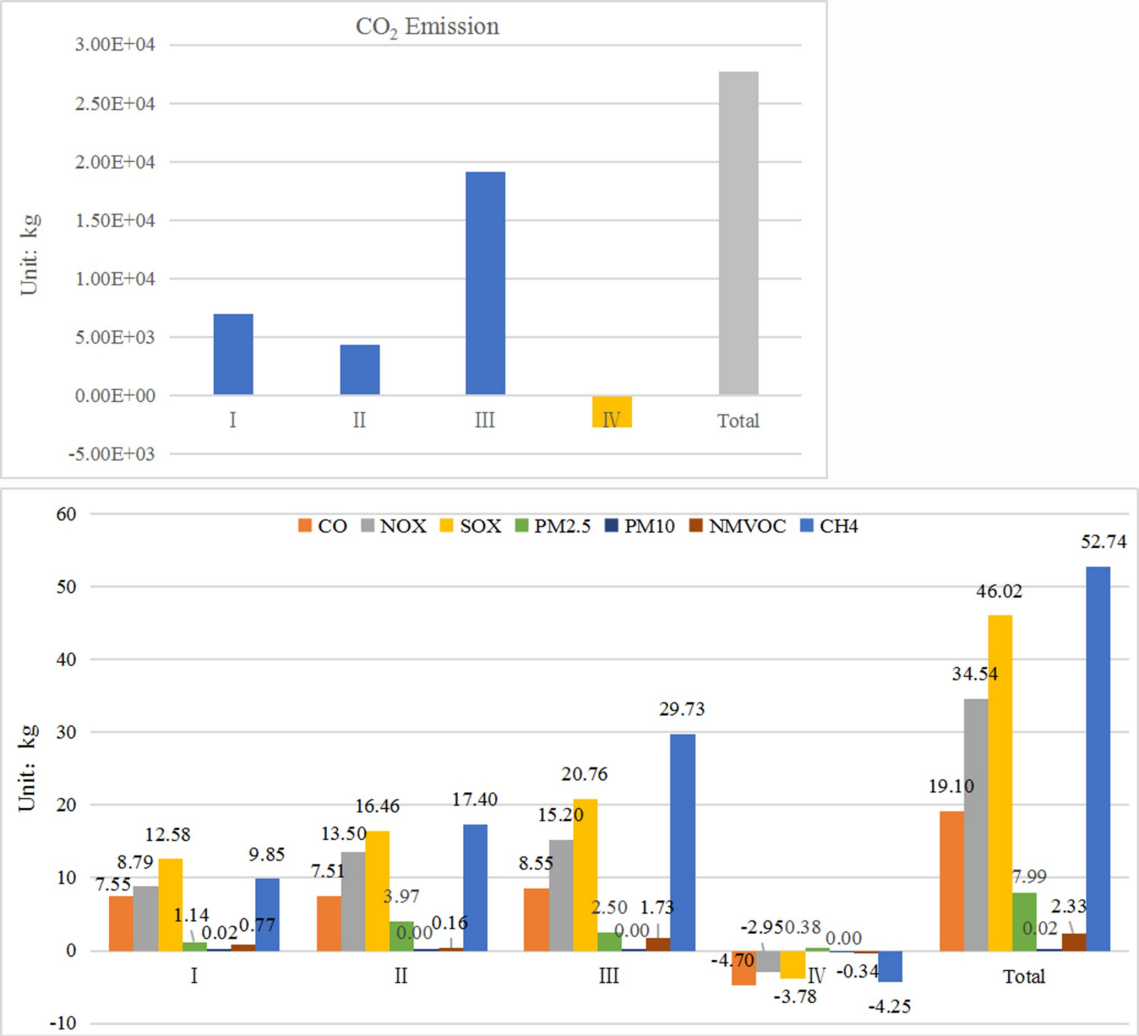
Pollutant emission at each stage of EREV life cycle (a and b). (Note: I—Raw material acquisition stage, II—Manufacturing and assembly stage, III—Operation and use stage, IV—Scrap recovery stage).

Fig 3 shows the calculation results of the emission intensity of the seven major emissions (CO_2_, CO, NO_X_, SO_X_, PM, NMVOC, and CH_4_). The four stages of extended range electric vehicles i.e., from the raw material acquisition stage to the scrap recovery stage, are accompanied by power consumption, especially the operation and use stage. At present, thermal power accounts for 72% of China's power structure, leading to more carbon dioxide emissions. From Fig 3(A), CO_2_ emissions mainly occur at the operation stage. At this stage, gasoline and electricity provide power for the EREV, and combustion and electricity generation result in large amounts of CO_2_ emissions. As can be seen from Fig 3(B), CH_4_ emissions mainly occur at the raw material acquisition stage, which is mainly produced by the mining and smelting processes. The PM analyzed in this paper mainly includes PM2.5 and PM10, which are mainly emitted at the operation stage and mainly caused by electricity production and automobile exhaust emissions. At the same time, mineral extraction and other processes at the raw material acquisition stage also result in more particulate emissions. The emissions of CO, NO_X_, SO_X_ and NMVOC mainly occur at the operation phase, and their proportions exceed 50% of the total emissions during their life cycles. At this stage, the emissions are mainly generated by gasoline production, electricity generation and other processes, while the direct emissions of vehicles only account for a small proportion and can be basically neglected. At the disposal and recycling stage, some emissions are negative. This is because the recycling of metal materials eliminates the mining, smelting, and other steps for the raw materials, effectively reducing the emissions at raw material production stage. As we all know, thermal power generation has an important impact on environmental emissions. Therefore, reducing the share of thermal power generation by applying advanced desulphurization technologies and using cleaner fuels for electricity generation is important to reduce the emissions of various pollutants. Meanwhile, in the process of gasoline production, the advanced petroleum mining and smelting technology can reduce the emissions of SO_X_, NO_X_ and other gases and improve oil quality and fuel efficiency.

#### 3.1.2 Characterization and normalization

According to the CML2001 evaluation method, the inventory results (mineral resource consumption, energy consumption and environmental emissions) were classified, converted and summarized into a unified unit and dimension. In other words, the results were characterized [38].

**Fig 4:**
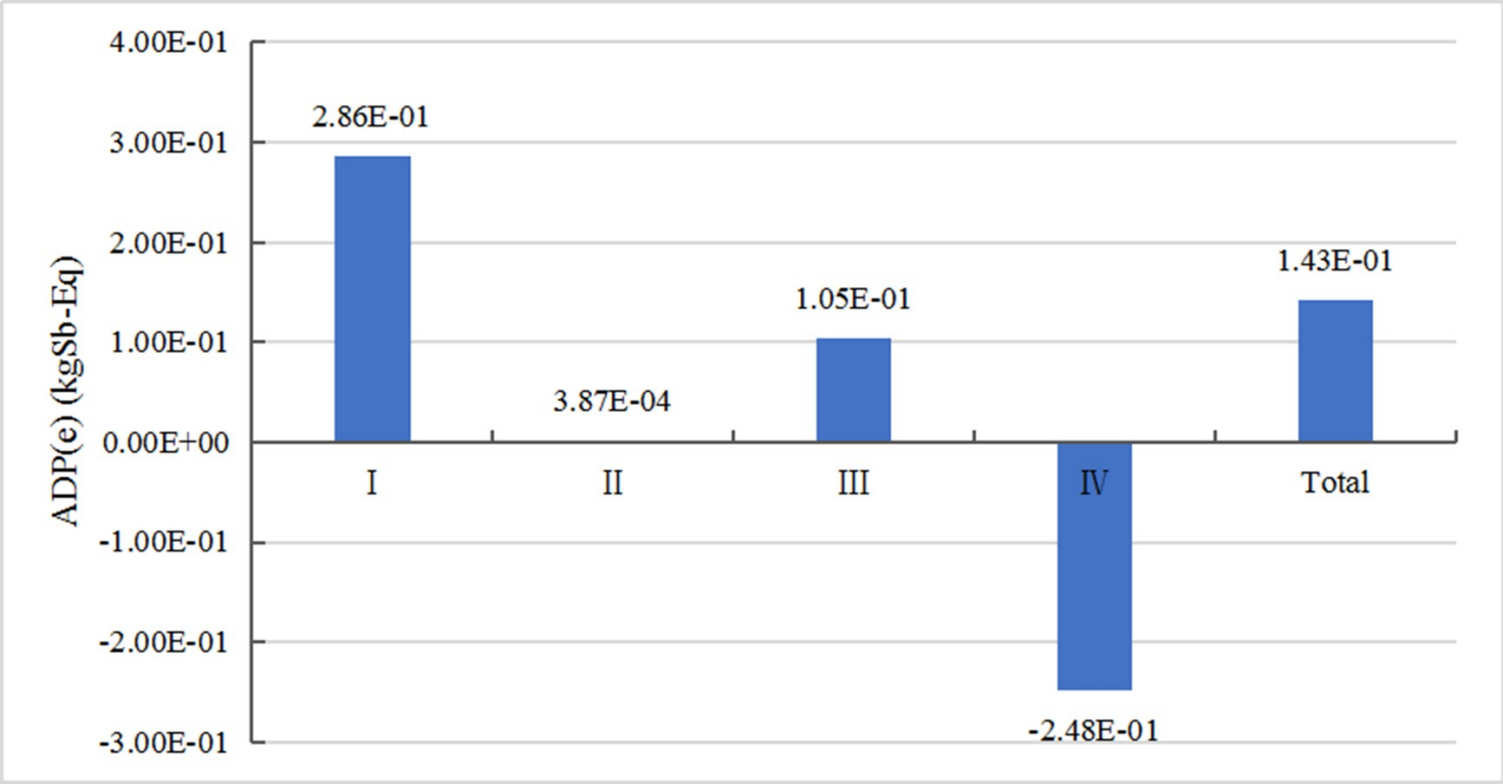
Consumption of mineral resources at each stage of EREV life cycle.

*1) Analysis of mineral resource consumption*. Fig 4 shows the mineral resource depletion potential (ADP (e)) at various stages. In the indicator characterization process, metal antimony (Sb) with the unit of kg Sb-eq was used as the reference substance. It can be found that at the raw material acquisition stage, the consumption of mineral resources is the highest, because the manufacture of vehicles requires a large amount of metals such as iron, copper, and aluminum. At manufacturing and assembly stage, the consumption of mineral resources is mainly caused by the welding and the use of some parts. At the operation stage, the consumption of mineral resources mainly comes from the regular replacement of batteries, tires, brake pads and other parts. At the disposal and recycling stage, ADP (e) is negative, because most of the metal materials of the vehicle are easier to recycle and have higher recycling rate than other materials. Therefore, the disposal and recycling of the vehicle generates considerable benefits for the depletion of mineral resources. In the future, with the improvement of recycling and reuse technologies, the benefits of this stage may further increase.

**Fig 5:**
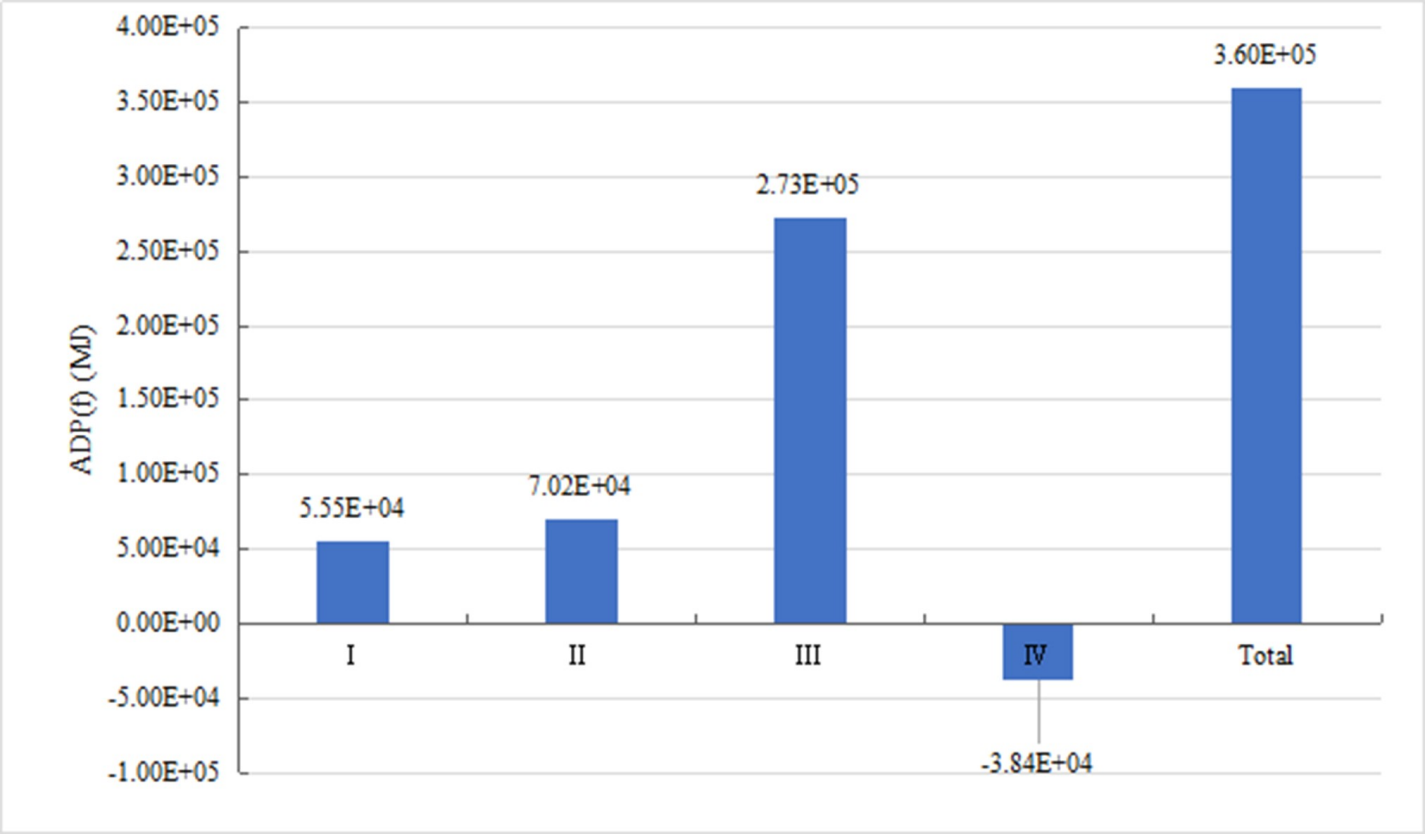
Fossil energy consumption at each stage of EREV life cycle.

*2) Analysis of fossil energy consumption*. Fig 5 shows the fossil energy depletion potential (ADP (f)) at various stages. The unit of ADP (f) is MJ. It can be observed that most of fossil energy is consumed at the operation stage. This is mainly due to the presence of engine and power battery in the extended range electric vehicle, which consumes a large amount of gasoline and electricity throughout operation and use stage (150,000km). The second highest consumption occurs at the manufacturing and assembly stage. The main reason is that all parts in the manufacturing and assembling process need to consume electric energy and heat energy, and the upstream of electric energy and heat energy will consume fossil energy. At the raw material acquisition stage, the mining process of minerals, raw coal, etc. consumes a lot of crude oil, and the metal material smelting process also leads to energy consumption. The energy consumption at the disposal and recycling stage produces positive benefits. Part of energy consumption can be saved by recycling metal materials. As the regeneration rate of metal materials increases, the energy saving can be significantly improved.

**Fig 6:**
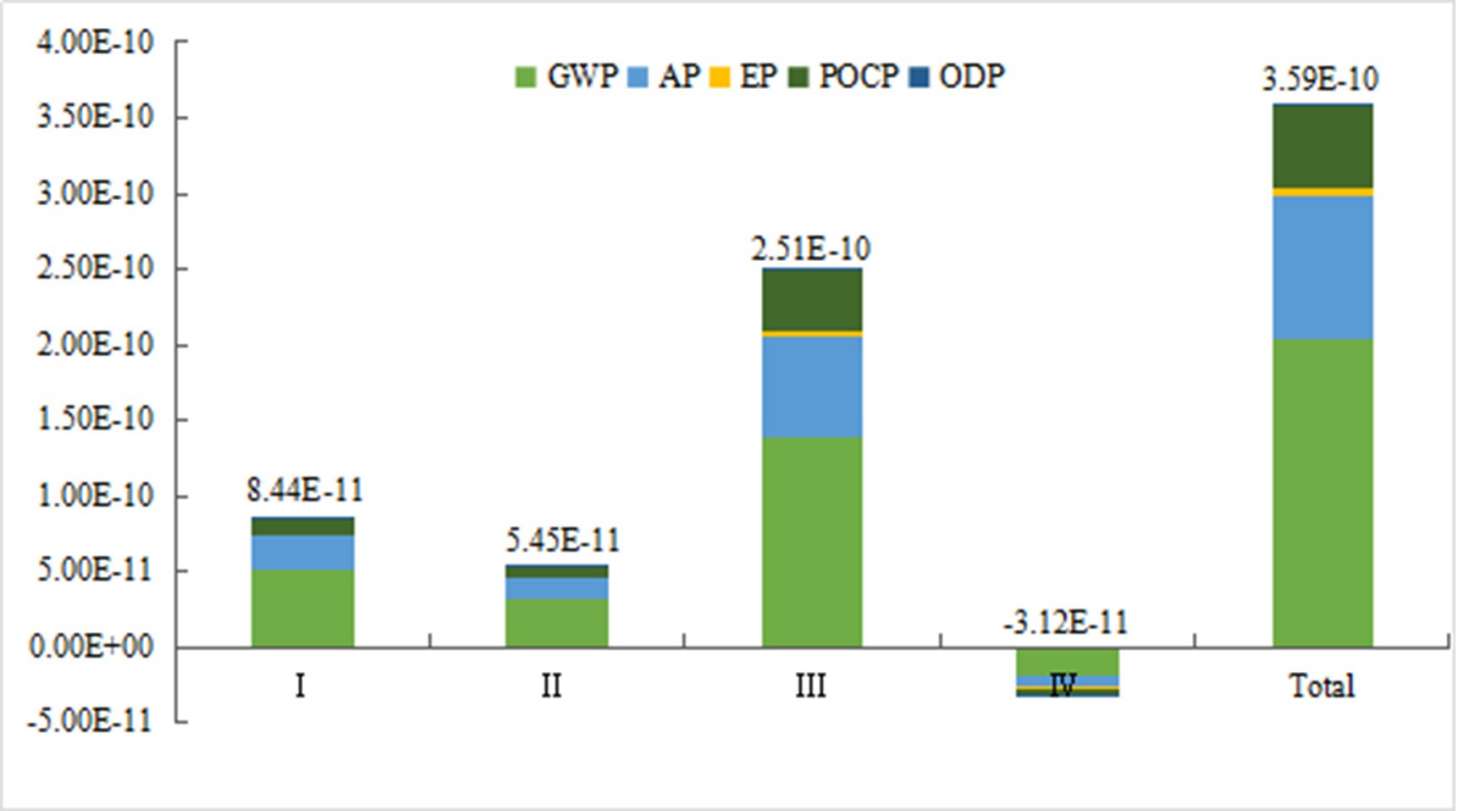
Integrated environmental impact at each stage of the life cycle.

*3) Analysis of environmental emission impact*. In order to investigate the importance of various environmental impact types, CML2001 was used to normalize and quantize the above environmental impact types. The normalized reference value was obtained from the GaBi database, and the weight coefficient was obtained from Literature [39]. The normalized reference value and the weight coefficient are shown in Table 2. The normalized and quantified results of the five environmental impact types are shown in Fig 6.

**Table 2:** Normalized reference value and weight coefficient.

Types of environmental impact	Normalized reference value and unit	Weight coefficient
Global warming potential(GWP)	4.18E+13 kg CO_2_-Eq	0.27469229
Acidification potential (AP)	2.39E+11 kg SO_2_-Eq	0.18072651
Eutrophication potential (EP)	1.58E+11 kg Phosphate-Eq	0.0888622
Photochemical smog potential (POCP)	3.68E+10 kg Ethene-Eq	0.18102672
Ozone layer depletion potential (ODP)	2.27E+08 kg CFC-Eq	0.27469229

In the four life cycle stages, the five types of environmental impact can be sorted from high to low as follows, GWP (56.5%)>AP (26.4%)>POCP (15.6%)>EP (1.45%)>ODP (0.05%). Fig 6 shows that the environmental impact of EREV is mainly at the operation stage, followed by the raw material acquisition stage and the manufacturing and assembly stage. At the operation stage, the main environmental impacts include GWP, AP and POCP. The POCP comes from the large amount of NO_X_ emissions, the AP is caused by the production of SO_2_ during the combustion process, and the GWP is due to the large amount of CO_2_ in the exhaust emissions. Therefore, it is necessary to reduce the greenhouse gas emissions from gasoline combustion and power production. At the raw material production stage, the production of aluminum alloy, steel and other raw materials consumes a lot of fossil energy, thereby producing a large amount of greenhouse gases, acidified gases, and nitrogen oxygen compounds.

### 3.2 Differential evaluation of vehicles with different power sources

#### 3.2.1 Analysis sample

In order to compare the life cycle resource consumption and environmental emissions of EREVs and other vehicles with different power sources, we have selected five types of cars, i.e., BEVs, HEVs, PHEVs, FCVs, ICEVs, which have the same size as EREVs. Table 3 shows the comparison of the basic parameters of these six types of vehicles.

**Table 3:** Basic parameters and comparison of six vehicle models with different power sources (collected from public data).

Vehicle Type	Whole vehicle mass (kg)	Maximum speed (km/h)	Engine capacity (L)	Power			Energy storage			Energy consumption			Recharge mileage (km)	
				Engine power (kw)	Motor Power (kw)	Generator (kw)	Power battery capacity (kWh)	Fuel tank capacity (L)	Hydrogen tank volume (L)	Fuel Consumption (L/100km)	Electric power consumption (kwh/100km)	Hydrogen consumption (L/100km)	Pure electric	Fuel
EREV	1700	160	1.4	63	111	55	16	35	∕	1.6	13	∕	64	490
BEV	1800	150	∕	∕	110	∕	60	∕	∕	∕	16	∕	410	∕
HEV	1350	180	1.2	85	∕	∕	∕	50	∕	4.3	∕	∕	∕	500
PHEV	1535	165	1.8	73	53	∕	10.5	43	∕	1.84	12	∕	55	560
FCV	1850	175	∕	∕	113	∕	∕	∕	122.4	∕	∕	0.85	650	∕
ICEV	1258	190	1.6	102	∕	∕	∕	47	∕	5.3	∕	∕	∕	600

The calculation model in Section 2.4 was used to calculate the differences in resource depletion and environmental impact between EREV and other five vehicles (BEV, HEV, PHEV, FCV, and ICEV) throughout the life cycle.

#### 3.2.2 Differences in resource depletion

**Fig 7:**
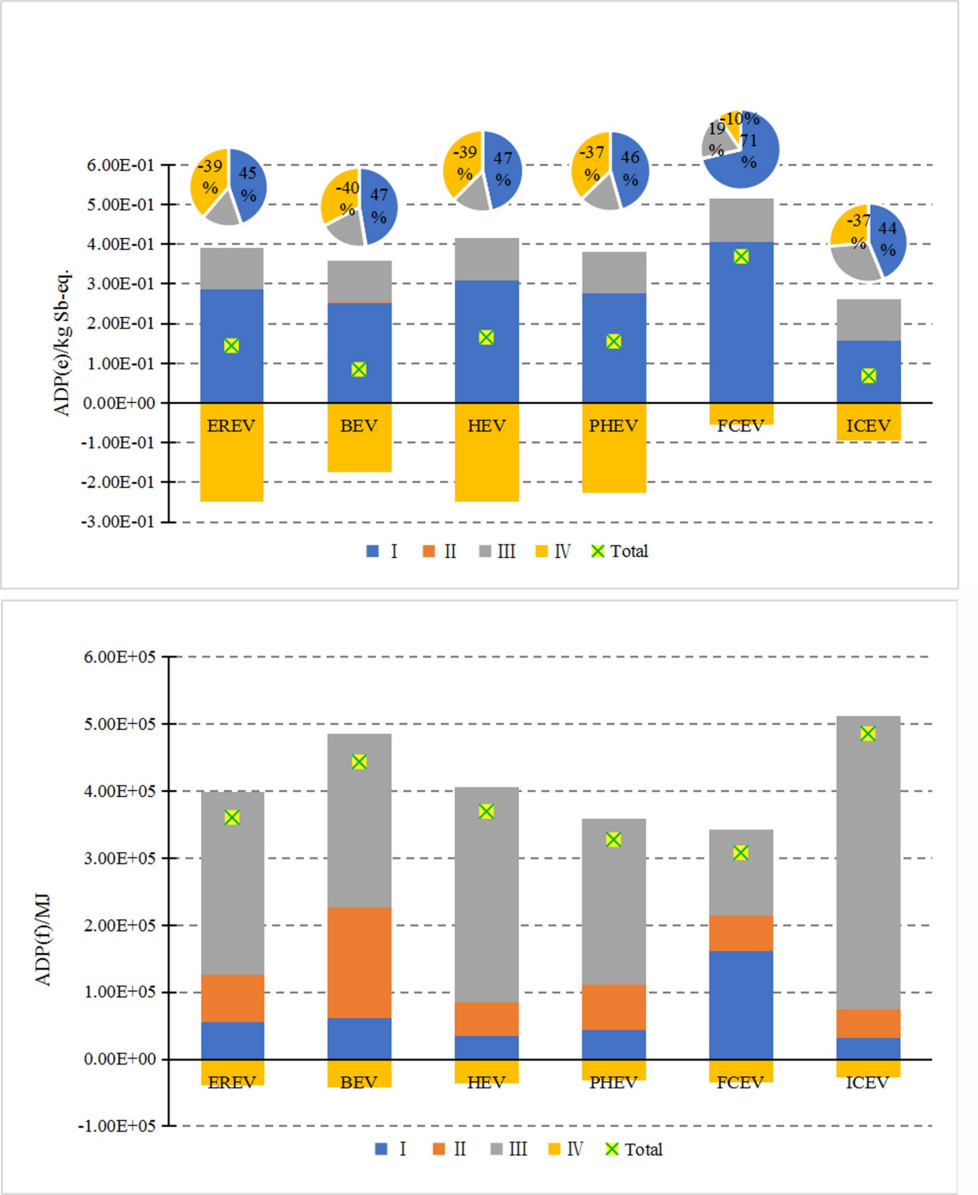
Difference of ADP of six vehicles with different power systems throughout the life cycle (a and b) (the part less than 1% is not shown).

Fig 7(A) shows the differences in ADP (e) among these six types of vehicles. For all the compared vehicles, the consumption of mineral resources is the highest at the raw material acquisition stage, and the disposal and recycling play an important and positive role in saving mineral resources and reducing environmental impact. Among all the vehicles, the consumption of mineral resources is mainly concentrated in the raw material acquisition stage, with FCV accounting for the largest proportion, mainly because platinum is used as catalyst in fuel cell engine. The extraction and manufacturing of the platinum metal consume a large amount of ore. The three hybrid electric vehicles, i.e., EREV, PHEV, and HEV only have little difference in performance. ICEV has simpler vehicle structure and does not contain power battery, fuel cell stacks or other components that consume a large amount of rare metals. Thus among these six types of vehicles, ICEV has the smallest ADP (e), followed by BEV. At the disposal and recycling stage, the positive benefits of mineral resources and environmental impact of FCV vehicles are relatively low, mainly because the current recycling process for the FCV is immature and the recycling rate for rare metals in the battery stack is low.

Fig 7(B) shows the difference in ADP (f) among the six types of vehicles. Overall, for all the six types of vehicles, the fossil energy is mainly consumed at the operation stage, and the positive benefits are produced at the disposal and recycling stage. ICEV has the highest consumption, because the traditional vehicles consume a large amount of fossil energy such as gasoline during the entire operation period. BEV has the second highest consumption. For BEV, the manufacturing of power batteries and other parts consume a lot of electric/thermal energy, resulting in the high consumption of fossil energy. In addition, although BEV does not directly consume fuel, it needs to be charged during the operation stage. In China, fossil fuels are used as the main source of power generation. Therefore, a lot of fossil energy is consumed by BEV during the operation stage. The consumption of the three hybrid electric vehicles can be sorted from large to small as follows: HEV > EREV> PHEV. HEV is equipped with a large-displacement engine, leading to more life cycle fossil energy consumption. For EREV and PHEV, the fossil energy consumption during the operation stage is lower than ICEV and HEV, and close to BEV. Therefore, EREV and PHEV have the obvious advantages in reducing fossil energy consumption.

#### 3.2.3 Differences in environmental impact

**Fig 8:**
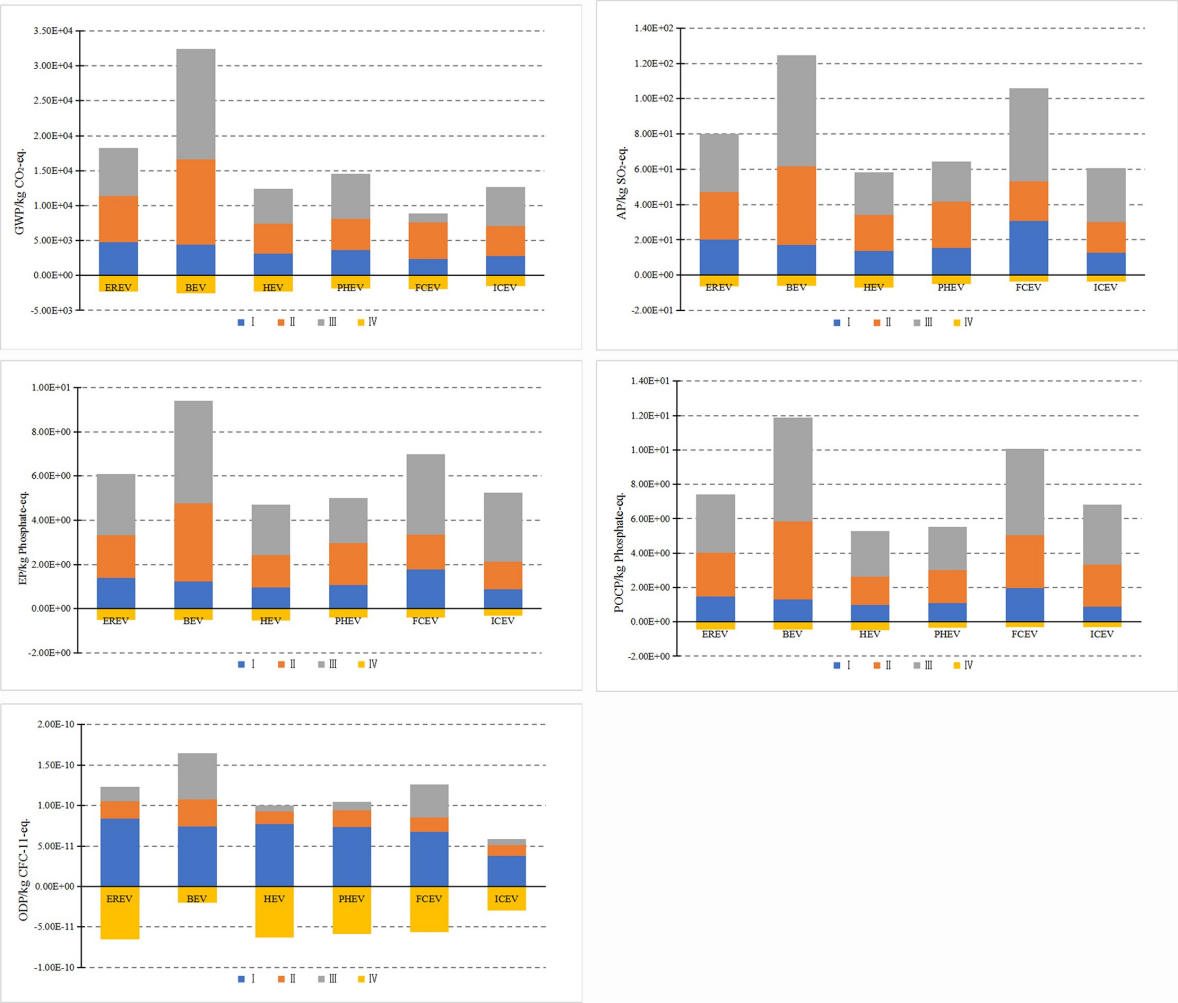
Environmental impact differences of six vehicles with different power systems throughout the life cycle. (a). Difference of GWP. (b). Difference in AP. (c). Difference in EP. (d). Difference of POCP. **(**e). Difference of ODP.

Fig 8(A) shows the difference in greenhouse effect emissions of these six types of vehicles. BEV causes the highest greenhouse effect, which is about 1.8 times as high as that from an EREV. The reason is that BEV need to be equipped with power batteries with higher energy density, which increases the vehicle's curb weight and results in higher power consumption per 100 km in the operation stage. However, the power structure in China is mainly thermal power generation. The greenhouse gases generated from coal combustion are directly emitted into the air, which intensifies the greenhouse effect. FCV has the lowest greenhouse effect, which is about 23.1% of the greenhouse effect from BEV. During the operation stage, FCV consumes the hydrogen and oxygen, and emits the water with zero emissions. The three hybrid vehicles, i.e., an EREV, HEV and PHEV, have a greenhouse effect of about 1.43 times, 0.91 times and 1.14 times as high as ICEV, respectively.

Fig 8(B) shows the difference in the acidification potential. The acidification potentials of BEV and FCV are relatively high, close to 1.62 times and 1.38 times as high as that of EREV, respectively. BEV consumes a lot of electrical energy, whose upstream energy source is mainly coal. The combustion of coal produces a large amount of acidic emissions such as SO_2_ and NO_2_. The high acidification potential of FCV is mainly due to the production of sulfur oxides and nitrogen oxides during the hydrogen production process. The acidification potentials of the three hybrid vehicles, i.e., an EREV, HEV and PHEV, are about 1.28 times, 0.89 times and 1.04 times as high as that of ICEV, respectively.

Fig 8(C) shows the difference in the eutrophication potential. The BEV has the most significant impact, followed by the FCV. BEV uses the lithium iron phosphate battery and produces nitrogen, phosphorus and other compounds during the production stage and the disposal and recycling stage. The eutrophication potentials of the three hybrid vehicles, i.e., an EREV, HEV and PHEV, are 1.13 times, 0.84 times and 0.94 times as high as that of ICEV, respectively.

Fig 8(D) shows the difference in photochemical smog potential. BEV has the most significant impact. BEV consumes a large amount of electrical energy from coal power generation, generating non-methane hydrocarbons, NO_X_ and CO emissions. The photochemical smog potentials of the three hybrid vehicles, i.e., an EREV, HEV and PHEV, are about 1.85 times, 0.78 times and 0.83 times as high as that of ICEV, respectively.

Fig 8(E) shows the difference in ozone depletion potential. BEV has the most significant influence. This is due to the great mass of the power battery, in which leads to a large vehicle mass and high power consumption. The ozone depletion potentials of EREV and FCV are close. The ozone depletion potentials of the three hybrid vehicles, i.e., an EREV, HEV, and PHEV, are about 1.96 times, 1.25 times, and 1.55 times as high as that of ICEV, respectively.

**Fig 9:**
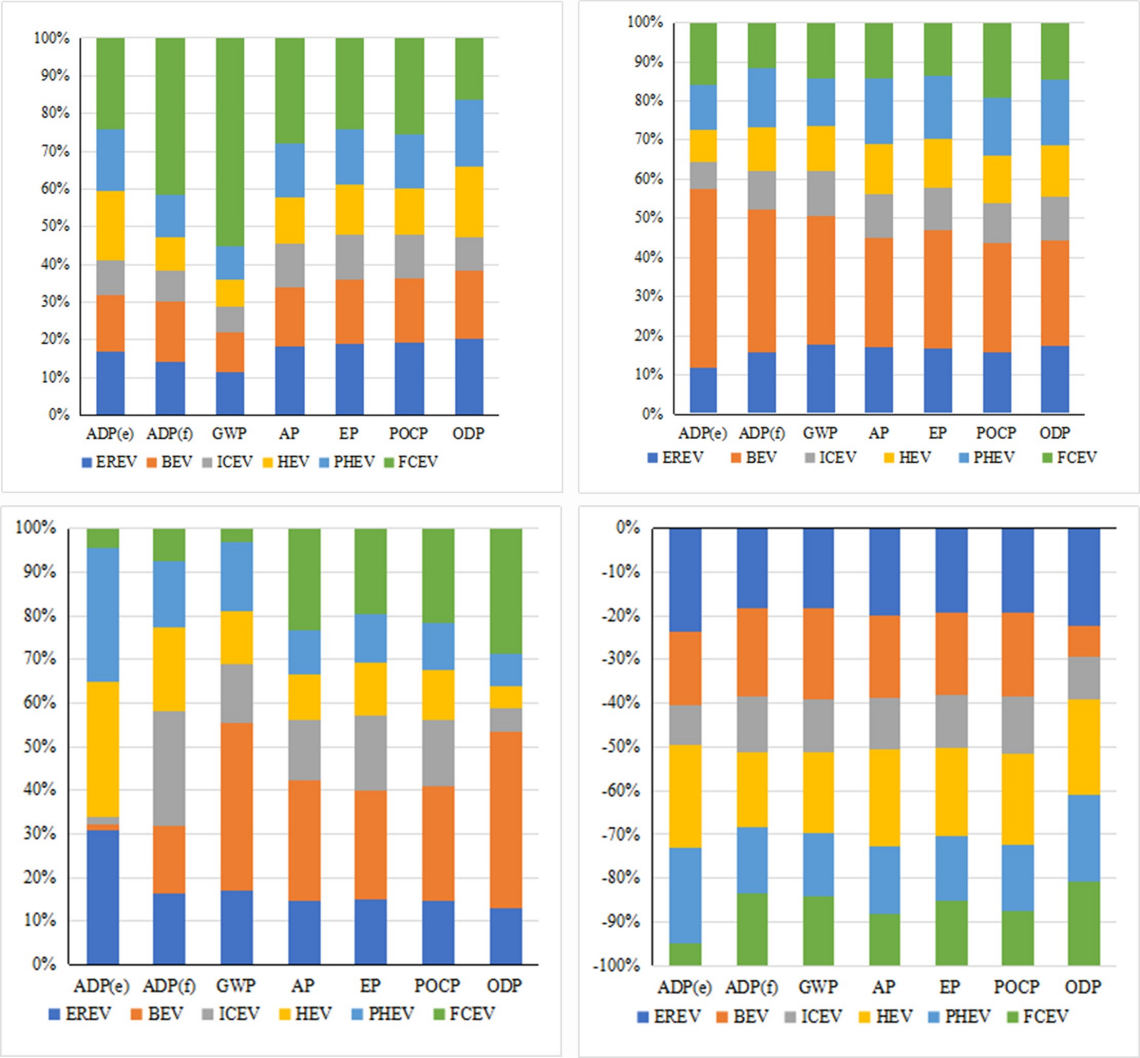
Impact difference of energy consumption and emission of six vehicles with different power systems during different stages. (a). Raw material acquisition stage. (b). Manufacturing and assembly stage. (c). Operation and use stage. (d). Scrap recovery stage.

Fig 9(A)–9(D) show the proportions of resource consumption, energy consumption, and environmental impacts of the six types of vehicles at various stages. From Fig 9(A), at the raw material acquisition stage, the consumptions of mineral resource ADP (e) and fossil energy ADP (f) are positively correlated with the degree of electrification of the car. In addition, at this stage, FCV has the highest environmental impact. EREV and BEV have the second highest environmental impact, and ICEV has the lowest environmental impact.

As shown in Fig 9(B), at the manufacturing and assembly stage, BEV has higher energy consumption and environmental impact than the other five types of vehicles. The reason is that the manufacturing and assembly process of power battery consume more energy. Fig 9(C) shows that, at the operation stage, there is a significant difference among six types of vehicles due to the different power sources. ICEV has the largest proportion of fossil energy consumption. BEV has the largest portion of greenhouse effect. Fig 9(D) indicates that, during the disposal and recycling stage, all these six vehicles produce significantly positive benefits in reducing resource consumption and environmental impact.

### 3.3 Scenario analysis

#### 3.3.1 Different vehicle sizes

This section further discusses the most suitable vehicle size of EREV in China from two aspects, i.e., life cycle resource consumption and environmental impact. According to the classification of motor vehicles from the Ministry of Public Security GA802-2014 standard, in this work, a total of twelve light-duty trucks and heavy-duty trucks were used as the research models. They had six different sizes, similar performance, and different power types (EREV, BEV, HEV, PHEV, FCV, and ICEV). Among them, the traditional light trucks and traditional heavy trucks are diesel vehicles. Due to the limitations of technology and application conditions, some models of the 12 trucks in this study do not exist, such as extended-range light trucks and extended-range heavy trucks. For these two models, this paper makes inference assumptions based on different models with the same power source and similar models with different power sources.

**Fig 10:**
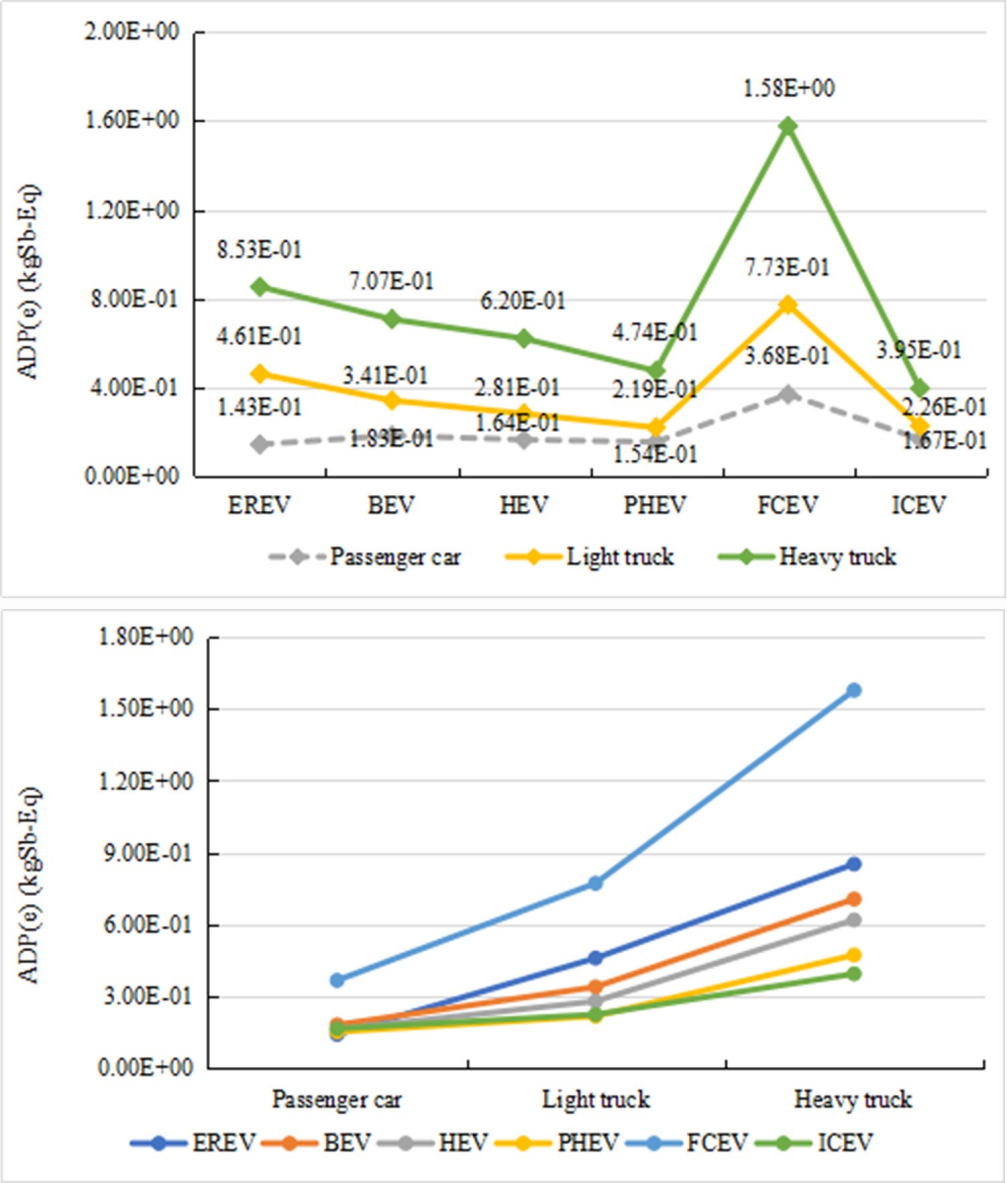
Differences in mineral resource consumption of vehicles of different sizes (a and b).

Fig 10 shows the differences in the mineral resource consumption of the six types of vehicles with different power systems and different sizes. From Fig 10(A), as the vehicle size increases, the mineral resource consumption tends to increase. The reason is that the increase in the vehicle mass results in more raw material consumption during the production stage. Among all the vehicles with the same size, FCV consumes the most mineral resources, while ICEV always has a relatively low ADP (e) value. It should be noted that for a passenger vehicle, the ADP (e) value of EREV is lower than that of BEV, HEV, and PHEV. However, for a light truck and a heavy truck, EREV has higher ADP (e) value than BEV, HEV, and PHEV. Fig 10(B) reveals that, when the vehicle size varies from the passenger car to the light truck, the corresponding change of ADP (e) value are different for vehicles with different power sources. PHEV and ICEV have the lowest sensitivity. And the sensitivity of other three types of vehicles can be sorted from high to low as follows: EREV> BEV > HEV. When the vehicle size changes from the light truck to the heavy truck, the change rate of EREV, BEV, and HEV vehicles almost remains the same, while the change rate of an FCV vehicle is higher than all other vehicles. As the size of ICEV changes, the ADP (e) value only has slight change.

**Fig 11:**
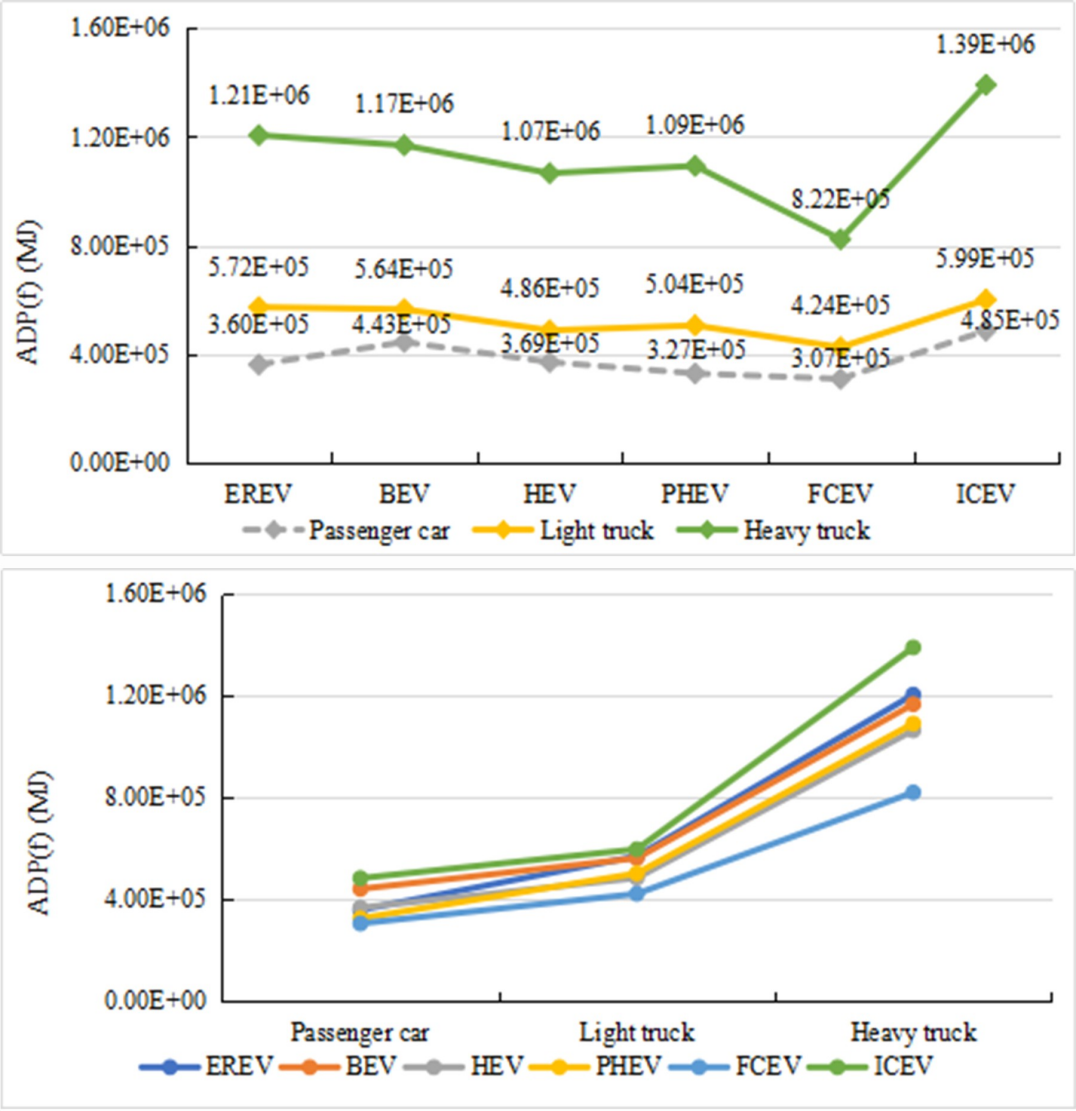
Differences in fossil energy consumption of vehicles of different sizes (a and b).

Fig 11 shows the differences in fossil energy consumption of six vehicles with different power systems and different sizes. From Fig 11(A), as the vehicle size increases, the overall fossil energy consumption increases. In addition, among the models with the same size, ICEV has the highest fossil energy consumption, while FCV has the least consumption. As the vehicle size increases, the energy saving effect of EREV gradually weakens. From Fig 11(B), when the vehicle model changes from a passenger vehicle to a light truck, the change rate of ADP (f) is the greatest in EREV vehicles, followed by PHEV. In other four types of vehicles, the change rate of ADP (f) is small and almost identical. When the vehicle size changes from a light truck to a heavy truck, the change rate of ADP (f) is the greatest in ICEV, followed by an EREV. As the vehicle size changes, ICEV's ADP (f) always remains the highest and is more sensitive to the size change. The reason may be that a larger vehicle uses a larger displacement engine, leading to an increase in the fossil fuel consumption during the life cycle.

**Fig 12:**
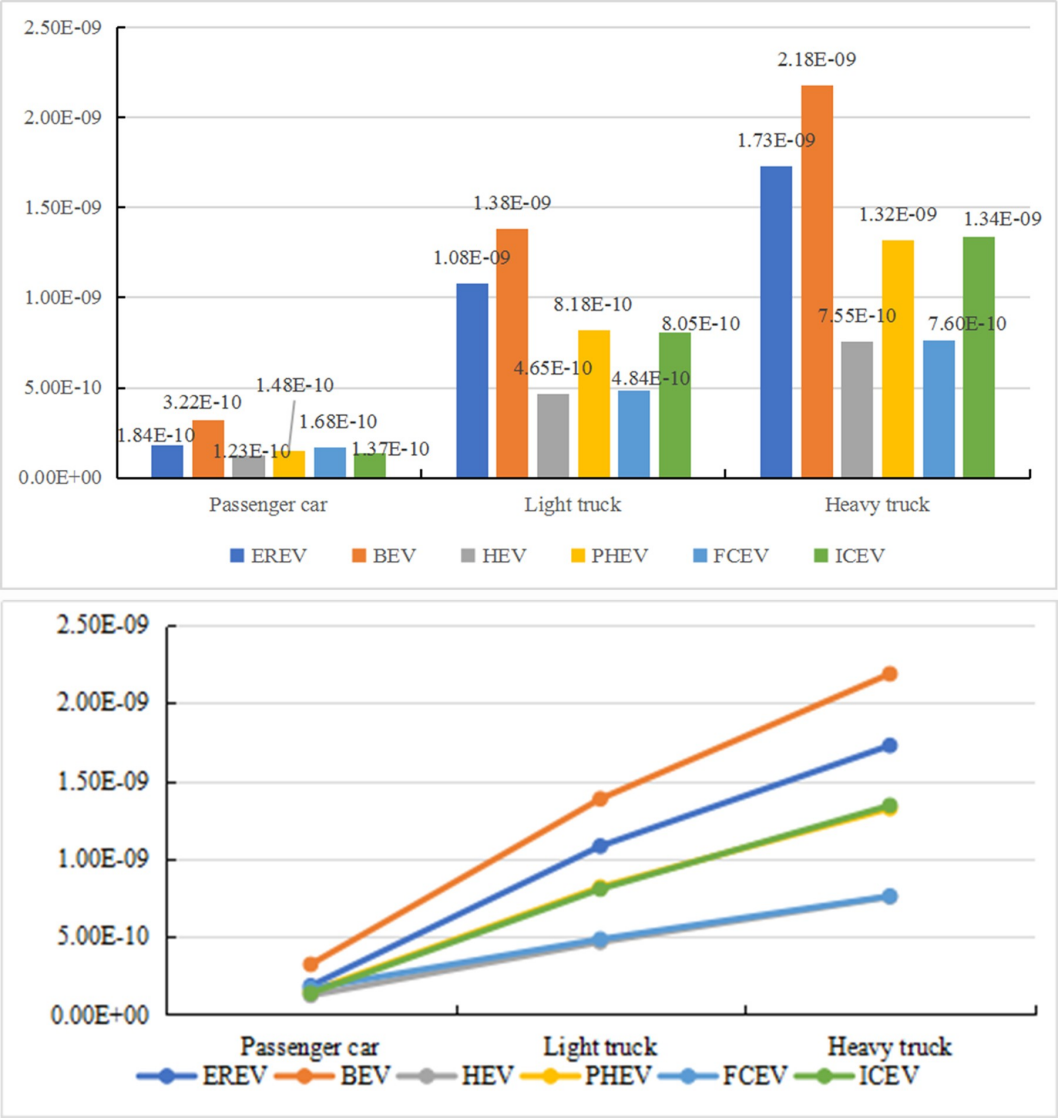
Difference of comprehensive environmental impact of vehicles of different sizes (a and b).

Fig 12 shows the differences in the life cycle environmental impact of six vehicles with different sizes and different power systems. From Fig 12(A), when the six types of power vehicles are passenger cars, light trucks, and heavy trucks, their environmental impacts are in the same trend. The BEV trucks have higher environmental impact than the other models. However, when the vehicle type changes from passenger car to truck, the REEV undergoes the most significant change in the environmental impact. For REEV, the environmental impact of heavy trucks is 1.60 and 9.40 times as high as that of light trucks and passenger cars, respectively. BEV has the second highest environmental impact. For BEV, the environmental impact of heavy trucks is 1.57 and 6.77 times higher than that of trucks and passenger cars. PHEV and ICEV have third highest environmental impact, and HEV and FCV perform better in terms of environmental impact. From Fig 12(B), the environmental impact of BEV is the most sensitive to the change of vehicle size, followed by an EREV. ICEV and PHEV have the same sensitivity. HEV and FCV have the small environmental impact, and are insensitive to the change of vehicle size.

#### 3.3.2 Different driving conditions

In order to explore the energy-saving and emission-reduction performance of the vehicles with six different power types under different driving conditions, this section compares three driving conditions, i.e., urban, comprehensive and suburban driving conditions. For some vehicle models, it is difficult to obtain fuel/electricity consumption data, thus the data from existing same-level vehicle models with similar performance were used in the calculation. At the operation stage, the main differences between vehicles stem from the structure of the power system. Thus the fuel/electricity consumption was mainly considered, while the consumption of mineral resources due to the replacement of tires and fluids was not considered in this paper. Therefore, this section mainly compares the fossil energy consumption and comprehensive environmental impact of the six types of vehicles under the combined, urban, and suburban conditions to obtain the best application scenarios for each vehicle model.

**Fig 13:**
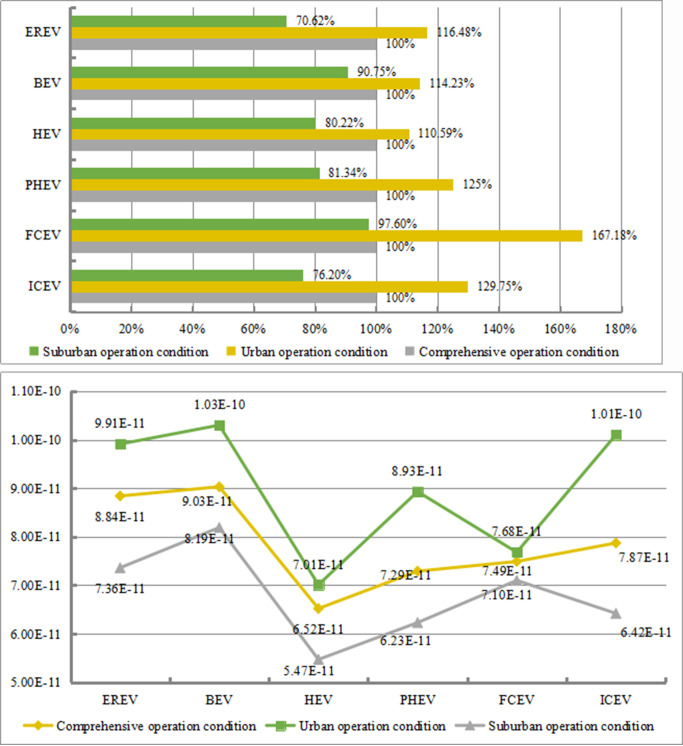
Comparison of the vehicles with six different power types in urban and suburban application scenarios. (a). Differences in fossil energy consumption. (b). Differences in comprehensive environmental impact.

From Fig 13(A), the differences in fossil energy consumption under the three driving conditions varies for the six models. The fossil energy consumption under the urban condition is higher than that under the combined condition and suburban condition. Among the vehicles, FCV has the most obvious difference among the three driving conditions. Under the urban condition, its fossil energy consumption is 67.18% and 69.58% higher than that under the combined condition and suburban condition, respectively. This phenomenon may be due to the low maturity of the FCV technology. Under urban condition, the frequency of braking and starting-up is higher, increasing the consumption of hydrogen energy. In addition, driving at a lower average speed also increases the additional consumption of hydrogen energy. Under the suburban condition, vehicles operate at a medium speed and consume less hydrogen energy, resulting in a better energy-saving effect. Therefore, FCV has the largest differences in fossil energy consumption under different driving conditions, followed by ICEV. The fossil energy consumed by ICEV in the urban area is 29.75% and 53.55% higher than that in the combined and suburban areas. For PHEV, the fossil energy consumption in the urban area is 25% and 43.66% higher than that in the combined and suburban areas, respectively. For an EREV, the fossil energy consumption in the urban area is 16.48% and 45.86% higher than that in the combined and suburban areas, respectively. For HEV, the fossil energy consumption in the urban area is 10.59% and 30.37% higher than that in the combined and suburban areas, respectively. For PHEV and an EREV, the increase in fossil energy consumption under urban condition is more significant than that of HEV. The reason may be that as the hybrid degree increases, there is energy conversion between the two power systems, resulting in a low energy utilization rate and higher energy consumption. Compared with other five types of vehicles, BEV has a smaller difference between urban condition and combined/suburban condition. The fossil energy consumption under the urban condition is 14.23% and 23.48% higher than that under the combined condition and suburban condition, respectively.

Fig 13(B) reveals that the six different types of vehicles have various environmental impacts. Overall, the environment impact under the urban condition is higher than that under the combined condition, while the environment impact under the suburban condition is lower than that under the combined condition. Among all the vehicles, for ICEV, PHEV, and EREV, the differences in the environment impact between various driving conditions are obvious. Comparing with the other two driving conditions, the energy consumption of vehicles under the suburban condition is lower, leading to the reduction of the pollutants emitted by vehicles. In addition, under the suburban condition, there is a high energy utilization rate, which helps to reduce energy consumption and indirectly abate the environmental impact of energy production.

## 4. Conclusions

In this paper, the energy-saving and emission-reduction performance of the EREVs were investigated based on the full life cycle theory. In order to clarify the effect of energy saving and emission reduction of vehicles in different technology routes, as well as explore the suitable vehicle size and the suitable application scenarios of EREVs, a comparative analysis of six vehicles with different power systems was conducted. The following conclusions have been obtained:

1. For an EREV, the mineral resources are mostly consumed at the raw material acquisition stage. The fossil energy consumption is mostly consumed during the operation stage, accounting for approximately 75.6% of the total consumption. The disposal and recycling stage produce the obvious positive benefits, and the establishment of a standardized car recycling system may lead to better benefits for resources and energy savings. In addition, the magnitude of the environmental impact indicators can be sorted from high to low as follows: GWP (56.5%) > AP (26.4%) > POCP (15.6%) > EP (1.45%) > ODP (0.05%). The environmental impact of EREV is mainly at the operation stage, followed by the raw material acquisition stage and the manufacturing and assembly stage.
2. For all the vehicles under six different technical routes, the mineral resources is mainly consumed at the raw material acquisition stage, and the recycling of vehicles is important for saving mineral resources and reducing environmental impact. Compared with HEV and PHEV, EREV has great advantages in reducing mineral resource consumption and fossil energy consumption. In terms of environmental impact, EREV lies in the middle position, and BEV has a great impact on the environment. The poor environmental benefits of BEV are due to the large vehicle mass, the production and manufacturing processes of power batteries, and the unclear electrical power structure in China.
3. The suitable size of the vehicles with six different power systems has been investigated. In general, the mineral resource consumption increases with the increase of the vehicle size. Among all the vehicles with the same size, FCV consumes the most mineral resources, and the ADP (e) value of ICEV always remains relatively low. The fossil energy consumption has the same trend as the mineral resource consumption. However, among the vehicles of the same size, ICEV has the highest fossil energy consumption, and FCV has the least fossil energy consumption. As the vehicle size increases, the energy-saving effect of EREV gradually weakens. The six vehicles have mild differences in environmental impact for a passenger car. However, for a truck, the difference in the environmental impact among the six vehicles is significant. For a truck, BEV has the highest environmental impact, followed by EREV, HEV and FCV.
4. When comparing the three driving conditions, i.e., urban, combined and suburban, it has been found that for all the vehicles with six different types of power systems, the urban driving condition are superior to the combined condition and suburban condition. FCV has the largest difference between different driving conditions. For FCV, the fossil energy consumption under the urban condition is 67.18% and 69.58% higher than that under the combined condition and suburban condition, respectively. ICEV has the second largest difference. For ICEV, the fossil energy consumption under the urban condition is 29.75% and 53.55% higher than that under the combined condition and suburban condition, respectively. PHEV and EREV have larger differences than HEV, and BEV has the lowest difference. The environmental impact under the urban condition is higher than that under the combined condition, while the environmental impact under the suburban condition is lower than that under the combined condition. ICEV, PHEV, and EREV have more obvious differences in the environmental impact under different driving conditions.

In the future, during the full life cycle evaluation of the vehicle, the impact of energy consumption and emissions in the transportation process can be comprehensively considered to improve the evaluation result. In addition, the scenario analysis in this study was mainly conducted under three driving conditions. However, in recent years, with the changes in road traffic conditions in China, the three driving conditions have certain deviations in the test. Moreover, the study of energy consumption and emissions can also be improved by the comparing the test conditions in various countries.

## Supporting information

S1 File Data set. (XLSX)
